# Pharmacokinetics of Schizandrin and Its Pharmaceutical Products Assessed Using a Validated LC–MS/MS Method

**DOI:** 10.3390/molecules23010173

**Published:** 2018-01-15

**Authors:** Chi-Lin Li, Yung-Yi Cheng, Chen-Hsi Hsieh, Tung-Hu Tsai

**Affiliations:** 1 Institute of Traditional Medicine, School of Medicine, National Yang-Ming University, Taipei 112, Taiwan; a2233525@gmail.com (C.-L.L.); vininecheng@gmail.com (Y.-Y.C.); 2 Division of Radiation Oncology, Department of Radiology, Far Eastern Memorial Hospital, Taipei 220, Taiwan; 3 Faculty of Medicine, School of Medicine, National Yang-Ming University, Taipei 112, Taiwan; 4 Graduate Institute of Acupuncture Science, China Medical University, Taichung 404, Taiwan; 5 School of Pharmacy, College of Pharmacy, Kaohsiung Medical University, Kaohsiung 807, Taiwan; 6 Department of Chemical Engineering, National United University, Miaoli 36063, Taiwan

**Keywords:** bioavailability, pharmacokinetics, liquid–liquid extraction method, ultra-performance liquid chromatography-tandem mass spectrometry, traditional Chinese medicine

## Abstract

*Schisandra chinensis* has been used as an important component in various prescriptions in traditional Chinese medicine and, more recently, in Western-based medicine for its anti-hepatotoxic effect. The aim of this study was to develop a selective, rapid, and sensitive ultra-performance liquid chromatography-tandem mass spectrometry method for pharmacokinetic studies of schizandrin in rats. Liquid-liquid extraction was used for plasma sample preparation. A UHPLC reverse-phase C18e column (100 mm × 2.1 mm, 2 μm) coupled with a mobile phase of methanol-0.1% formic acid (85:15, *v*/*v*) was used for sample separation. A triple quadrupole tandem mass spectrometer was used to detect the analytes in the selected reaction monitoring mode. The linear range of schizandrin in rat plasma was 5.0–1000 ng/mL (r^2^ > 0.999), with a lower limit of quantification of 5 ng/mL. The method was validated with regard to accuracy, intra-day and inter-day precision, linearity, stability, recovery, and matrix effects in rat plasma, which were acceptable according to the biological method validation guidelines developed by the FDA. This method was successfully applied to a pharmacokinetic study after oral administration of 3 g/kg and 10 g/kg of *Schisandra chinensis* products, which yielded a maximum concentration of schizandrin of 0.08 ± 0.07 and 0.15 ± 0.09 μg/mL, respectively. A parallel study design was used to investigate the oral bioavailability of single compound of schizandrin and the herbal extract, the single compound of pure schizandrin (10 mg/kg, i.v.), pure schizandrin (10 mg/kg, p.o.), and the herbal extract of *Schisandra chinensis* (3 g/kg and 10 g/kg, p.o.) were given individually. The dose of *Schisandra chinensis* (3 g/kg) equivalent to schizandrin (5.2 mg/kg); the dose of *Schisandra chinensis* (10 g/kg) equivalent to schizandrin (17.3 mg/kg). The result demonstrated that the oral bioavailability of schizandrin was approximately 15.56 ± 10.47% in rats, however the oral bioavailability of herbal extract was higher than single compound. The method was successfully applied to the pharmacokinetic study of pure schizandrin after oral administration of its pharmaceutical industry products in rats.

## 1. Introduction

In traditional Chinese medicine, *Schisandra* fruit (Chinese herbal name: Wu-Wei-Zi) is a frequently used drug. According to the species of origin, clinically used two kinds of *Schisandra chinensis* were *Schisandra chinensis* and *Schisandra sphenanthera*. The active ingredients of the two *Schisandra* fruits have been analyzed, and their efficacy is comparable. In China and Japan, *Schisandra* fruit is mainly used to treat liver toxicity [1,2], asthma [3,4,5], and diabetes [6] and as a sedative and tonic [7,8]. *Schisandra* fruits are rich in dibenzocyclooctadiene-derived material. Botanical studies have shown that dibenzocyclooctadiene is the natural form. The presence of lignans is extreme low [9]. The 18-carbon dibenzocyclooctadiene lignans from *Schisandra* fruit have anti-oxidant [6,10,11], anti-hepatotoxic [12,13], and anti-lipid peroxidation [14] effects. It has been documented that the 18-carbon dibenzocyclooctadiene lignans have anti-human immunodeficiency virus effects [15,16], whereas homolignans containing 19 carbons are cytotoxic [17,18].

Recently, many Chinese medical doctors in Taiwan have come to prefer to prescribe commercial pharmaceutical herbal products instead of traditional decoctions because commercial pharmaceutical herbal products are convenient for patients. These products when produced by a cGMP pharmaceutical factory would be monitored for pesticide residues, heavy metals, and the dry herbs’ source, etc. However, because the manufacturing processes of the pharmaceuticals differ from factory to factory, the concentrations of active compounds may vary too. Therefore, it is important to determine the active compounds in each *S. chinensis* commercial pharmaceutical product.

Since the 1990s, much attention has been paid to the pharmacokinetics of schizandrin, considered the main active ingredient in *S. chinensis*, and several analytical methods have been used to analyze schizandrin in rats, including GC-MS [19,20,21,22], LC [23,24,25,26], TLC [9,27,28], LC-MS [29], high-speed counter current chromatography [30,31] and liquid chromatography tandem mass spectrometry (LC-MS/MS) [32]. LC-MS/MS has been applied to analyze the herbal ingredients in shengmai injection [32]. However, these methods are mostly applied to analyze raw herbs and extracts of *S. chinensis*. Samples are very time-consuming to obtain, with complicated sample pretreatments, and the methods are not sufficiently sensitive for pharmacokinetic studies. Although there is a previous study regarding the pharmacokinetics of orally dosing *S. chinensis*, its results were limited to giving comprehensive instructions for administering this herb. Herein, by investigating a conscious, freely moving animal model, it should more similar to the reality. To the best of our knowledge, there is no analytical method for the quantification of schizandrin and its pharmaceutical products in biological samples.

In recent years, LC-MS has been a ubiquitous technology for analyzing biosamples because its high sensitivity enables one to extend the concentration detection range. The aim of this study was to develop a convenient, specific and sensitive LC-MS/MS method for determination of schizandrin and its pharmaceutical industry products in biological samples. The validation and application of this assay are illustrated for the quantitative analysis of schizandrin in biological samples after oral administration of *S. chinensis* pharmaceutical products to rats. To investigate the oral bioavailability of the single compound schizandrin and the herbal extract, a parallel study design was followed using pure schizandrin (10 mg/kg, i.v.), pure schizandrin (10 mg/kg, p.o.), and *S. chinensis* (3 g/kg and 10 g/kg, p.o.). The 3 g/kg dose of *S. chinensis* was equivalent to 5.2 mg/kg schizandrin and the 10 g/kg dose of *S. chinensis* was equivalent to 17.3 mg/kg schizandrin.

## 2. Results and Discussion

### 2.1. Optimization of UPLC-MS/MS Conditions

**Figure 1:**
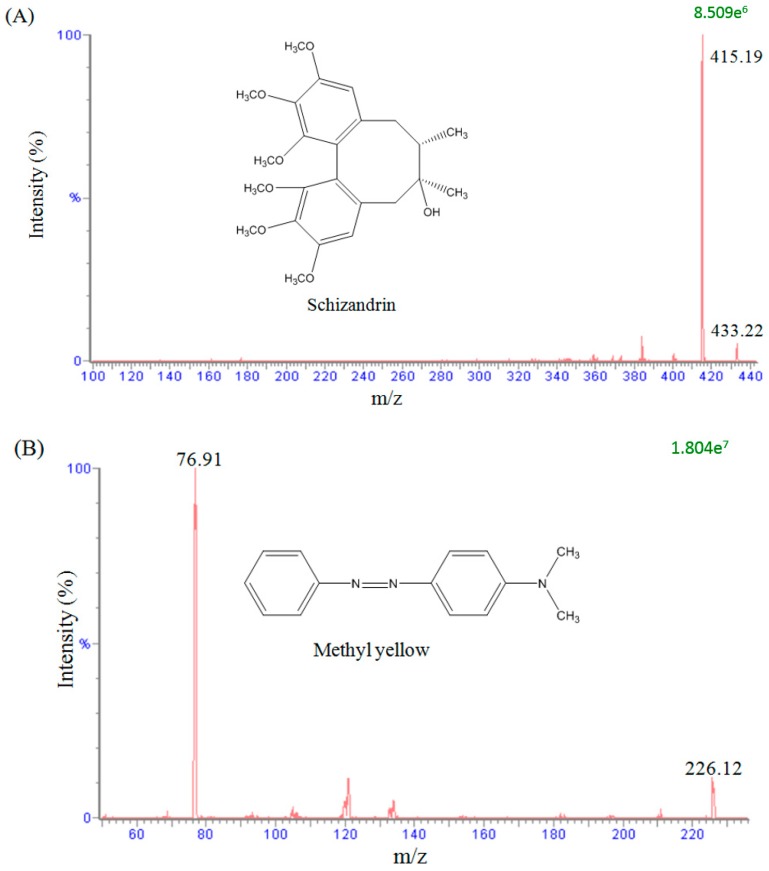
Representative product ion mass spectra and chemical structures of (**A**) schizandrin and (**B**) methyl yellow (internal standard).

To evaluate the mass spectral fragmentation patterns of schizandrin and to optimize the set of parameters used, a standard solution of schizandrin (100 ng/mL) was analyzed by direct injection in the spectrometer. In the analytical conditions, a full scan in the positive mode (scan range from *m*/*z* 100 to 500) was used to identify the analyte. The precursor ions of schizandrin and methyl yellow (used as internal standard) were at *m*/*z* 433.22 [M + H]^+^ and *m*/*z* 226.12 [M + H]^+^, and the main product ions were at *m*/*z* 415.19 and *m*/*z* 76.91, as shown in Figure 1. The analytes were detected in the positive ionization mode by monitoring the precursor-product combination in MRM mode, which provided high selectivity and sensitivity for the quantification assay.

**Figure 2:**
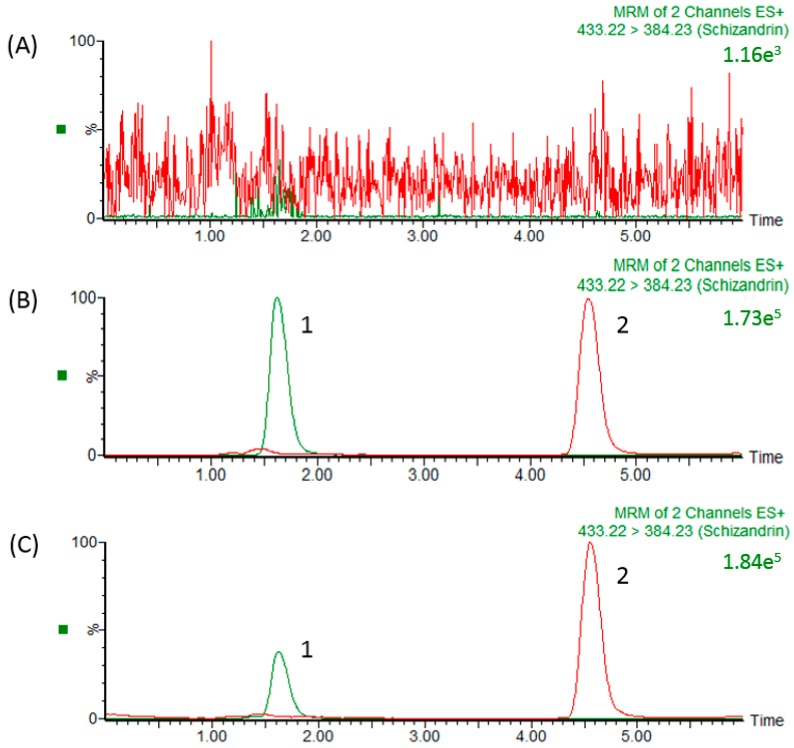
HPLC chromatograms (**A**) Blank plasma; (**B**) schizandrin standard (100 ng/mL) RT: 1.6 min; and (**C**) plasma sample collected from the jugular vein of a rat at 15 min after schizandrin (31.8 ng/mL) administration (10 mg/kg, i.v.). 1: Schizandrin; 2: methyl yellow (internal standard).

In our study, the precursor ion of schizandrin at *m*/*z* 433.22 [M + H]^+^ and its main product ion at *m*/*z* 415.19 were the same as those in a previous report [33]. After optimizing the assay conditions, the following experiments were performed to optimize the chromatographic separation of the analyte: chromatographic conditions, in particular the analytical column and the mobile phase composition (percentage of organic modifier, concentration and pH value of buffer), were optimized for good sensitivity and peak shape, as well as to achieve relatively short runs. It was observed that methanol produced a better peak shape than acetonitrile and was therefore selected as the organic phase. An improved peak shape was achieved by adding 0.1% formic acid to the mobile phase. Finally, a mobile phase consisting of a methanol-0.1% formic acid solution was used in the experiment. Under optimized conditions, the reaction chromatogram of each analyte is shown in Figure 2. Figure 2A shows chromatograms of blank plasma after liquid–liquid extraction, demonstrating the baseline without any interference peaks in the chromatograms of real samples. Figure 2B shows a chromatogram of a plasma sample spiked with schizandrin (100 ng/mL) and methyl yellow (10 ng/mL), and Figure 2C shows the real blood samples after schizandrin administration (10 mg/kg, i.v.) with methyl yellow (10 ng/mL).

### 2.2. Method Validation

Linear regression analysis of the calibration curve in blank plasma on six replicates per day over six different days indicate linearity between the nominal and the response concentration of schizandrin over the range of 5–500 ng/mL. The correlation coefficient (r^2^) was greater than 0.995. The data showed excellent reproducibility. The limit of quantification (LOQ) and limit of detection (LOD) were defined as the concentration of schizandrin detected as a signal-to-noise (S/N) ratio of 10 and 3, respectively. The LOQ and LOD of schizandrin were 5 and 1 ng/mL, respectively. The intra- and inter-day accuracy (% bias) and precision (% RSD) were determined at schizandrin concentrations of 5, 10, 50, 100, and 500 ng/mL. The results of these analyses are presented in Table 1, and all the bias and RSD values were within ±15%, except for the LLOQ, which was within ±20%, although it was still within an acceptable range. These results indicated that the UPLC–MS/MS method is excellent for the quantitative analysis of schizandrin in biological samples.

Three concentrations (low, medium, and high) were evaluated for recovery and matrix effects. The schizandrin concentrations detected were 5, 50, and 500 ng/mL, and the methyl yellow concentration was 10 ng/mL. The results indicated that the matrix effect and recovery of schizandrin in rat plasma were 91.5−97.8% and 90.8−99.6%, respectively (Table 2). There were no significant differences in the analytical range, which was acceptable according to the FDA’s biological method validation guidelines. The results indicated that this analytical method was acceptable for use in the pharmacokinetic study of schizandrin in freely moving rats.

The stability of schizandrin is shown in Table 3. Three concentrations (low, medium, and high) were evaluated for stability. The schizandrin concentrations of 5, 50, and 500 ng/mL were used in the method validation. The results demonstrated that schizandrin was stable under all the evaluated conditions. The schizandrin and IS stock solutions were found to be stable for up to 1 month at −20 °C. No significant degradation of schizandrin occurred in biological samples after autosampler storage for 24 h at 10 °C, after short-term storage for 12 h at room temperature, after long-term storage for 1 month at −80 °C, or after three freeze-thaw cycles at −20 °C and thawing at room temperature.

### 2.3. Sample Preparation

The sample preparation method was pretested and included a liquid–liquid extraction method (LLE) with ethyl acetate and protein precipitation with methanol [31]. Comparing the process efficiency of the LLE and protein precipitation, we found that protein precipitation had lower process efficiency due to the high polarity of schizandrin. Therefore, liquid–liquid extraction was selected as the sample preparation method in this study. The recovery (RE) and matrix effect (ME) were 90.8−99.6% and 91.5−97.8%, respectively. As shown in the above results, LLE with acidified ethyl acetate had a high efficiency with an acceptable RE and ME. In our study, LLE with acidified ethyl acetate was chosen as the sample preparation method.

### 2.4. Pharmacokinetic Applications

**Figure 3:**
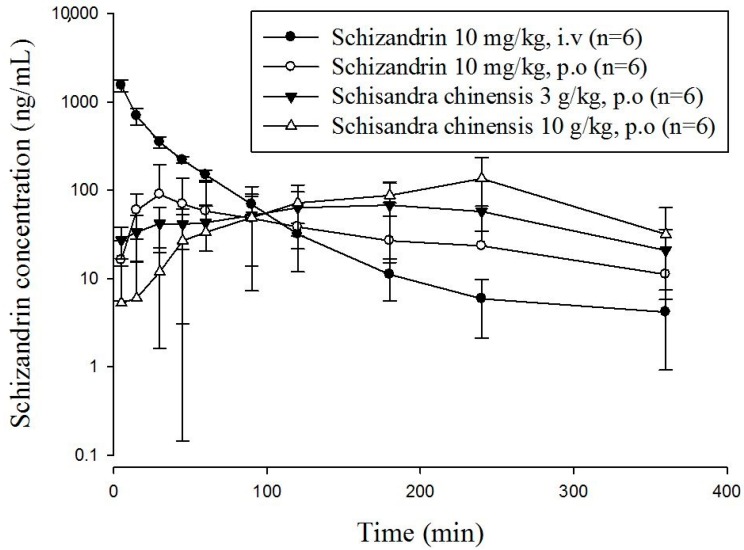
Concentration-time curve of schizandrin in rat plasma after intravenous (10 mg/kg, i.v.) and oral administration of *S. chinensis* (3 g/kg or 10 g/kg, p.o.) and schizandrin (10 mg/kg, p.o.); mean ± SD, *n* = 6.The dose of *S. chinensis* (3 g/kg) equivalent to schizandrin (5.2 mg/kg); the dose of *S. chinensis* (10 g/kg) equivalent to schizandrin (17.3 mg/kg).

This developed and validated method was used to determine the plasma concentration of schizandrin in freely moving rats. Because the pharmaceutical industry *Schisandra* products contained starch as the excipient, it was difficult to use a high concentration for oral gavage. Based on the pharmaceutical analysis, an oral single dose of *S. chinensis* (3 g/kg and 10 g/kg) was determined for the pharmacokinetic study in rats with a volume of 10 mL/kg [33]. The equivalent dosages of schizandrin of two oral *S. chinensis* product groups were 5.2 mg/kg for 3 g/kg and 17.3 mg/kg for 10 g/kg, respectively. The concentration-time profiles of schizandrin after oral administration of schizandrin or *S. chinensis* are presented in Figure 3.

The pharmacokinetic parameters are summarized in Table 4. The time to reach the maximum peak plasma concentration of a drug after administration in biological systems can be used as an index of the drug absorption rate. In our research, in oral administration groups, the T_max_ values range from 22 to 200 min. The basic pharmacokinetic and bioavailability information for schizandrin were obtained from a single intravenous treatment at one dosage (10 mg/kg) and oral administration at three dosages (10 mg/kg, 3 g/kg and 10 g/kg) in rats. Pharmacokinetic parameters were calculated by the software program WinNonlin.

Pharmacokinetic parameters for intravenous and oral administration were calculated by non-compartmental analysis, also called model independent analysis. The pharmacokinetic parameters of intravenous group revealed the half-life (t_1/2_), clearance (CL), mean residence time (MRT), area under the concentration-time curve (AUC) and volume of distribution (Vd). The pharmacokinetic parameter for MRT was 34.80 min, the CL was 0.09 L/min, and the AUC was 43.11 min ng/mL, suggesting that the elimination process is rapid. The areas under the concentration–time curves (AUCs) of ingesting schizandrin group was 6.71 ± 4.51, and other two groups were 17.58 ± 12.31 and 28.03 ± 14.29 min ng/mL for the dosages of 3 g/kg and 10 g/kg *S. chinensis* product, respectively.

Reviewing the profiles, up to 120 min, the plasma concentrations of schizandrin resulting from the pharmaceutical industry *S. chinensis* products were significantly higher than the concentrations resulting from orally administering the pure compound schizandrin. The clearances (CL) of the three orally administered at the dosages of 10 mg/kg, 3 g/kg, and 10 g/kg were 0.34 ± 1.24, 0.33 ± 0.24 and 0.30 ± 0.46 mL/min/kg, respectively. Although the T_max_ and the MRT of orally given schizandrin group were shorter than that of *S. chinensis* (*p* < 0.05) groups, there were no significant differences in the clearances among the three groups. The short half-life, short mean residence time and high clearance rate of schizandrin imply that the elimination process might be rapid. Our findings indicated that the T_max_ and MRT values of orally ingesting *S. chinensis* products are larger than that of the dosing single component schizandrin, which also matches Feary’s study [34]. According to previous reports, narcotics have an impact on the outcomes of animal experiments [35]. Therefore, the results might be different from those of the previous studies. We determined the bioavailability and the degree and rate at which a drug is absorbed into a circulatory system. Bioavailability was calculated using the following formula:

F (%) = 100 × (AUC_oral_/D_oral_)/(AUC_iv_/D_iv_).

In our study, we used the AUC_iv_ value of schizandrin to calculate the bioavailability of schizandrin and *S. chinensis* (3 g/kg and 10 g/kg). According to the data, the oral bioavailability of schizandrin was 15.56 ± 10.47%, and that of *S. chinensis* was 78.42 ± 54.91% and 37.59 ± 19.16%. The schizandrin data is the same as those in the previous report [36]. Hepatic first-pass and intestinal effects are widely acknowledged to be responsible for the low oral bioavailability of schizandrin. Systematic elimination may also occur in the gastrointestinal wall. Due to robust first-pass metabolism, schizandrin could be metabolized rapidly, resulting in less schizandrin entering the circulation and therefore presenting low bioavailability at high doses. Thus, schizandrin displays nonlinear pharmacokinetics after oral administration due to nonlinear metabolism in the liver. According to the comparative pharmacokinetics results shown in Table 4, the C_max_ and T_max_ of pharmaceutical industry *S. chinensis* products were significantly different from that of single ingestion group.

In Figure 3, the drug concentration of orally administering schizandrin reaches the C_max_ within 30 min, which was significantly faster and higher than those from given by *S. chinensis* product. This phenomenon may be due to the fact that pure compound schizandrin is a small molecule with a molecular weight less than 500 Da [37], which dissolves completely in water and did not form granules. Therefore, the absorption of pure compound schizandrin may be more efficient than its absorption from extracts containing mixtures of excipients. The schizandrin of the granulated pharmaceutical industry *S. chinensis* products were covered by excipients, which may release slowly and delay for absorption than pure compound. In addition, the other ingredients in *S. chinensis*, excipients in commercial pharmaceutical products, which usually contain starch for granulation, might affect the absorption of schizandrin. The schizandrin levels in the plasma of rats given the commercial pharmaceutical products showed a later T_max_ and longer persistence than the levels in rats given the pure compound. This effect may be due to the starch solution being mushy and prolonging the gastric emptying time. Based on those phenomena, we concluded that the plasma concentration of schizandrin in rats given *S. chinensis* was detected for 6 h, and the mean retention time was longer than that of the pure compound schizandrin, which might be because that schizandrin was reabsorbed into the blood and retained in the body. In this study, we first focused our investigation on the pharmacokinetic profile of major bioactive components in pharmaceutical products. The validated LC-MS/MS method was successfully applied to a pharmacokinetic study in freely moving rats after pure schizandrin and pharmaceutical products of *S. chinensis*. Finally, Traditional Chinese medicines have been used for thousands of years in Asia, and there is still a large amount of empirical information awaiting new scientific explanations. These studies provide constructive comparative pharmacokinetic information about single schizandrin and pharmaceutical industry products for clinical application

## 3. Materials and Methods

### 3.1. Chemicals and Reagents

Polyethylene glycol 400 (PEG 400) and heparin sodium were purchased from Sigma-Aldrich (St. Louis, MO, USA). Pentobarbital sodium was obtained from SCI Phar-matech (Toayuan, Taiwan). *S. chinensis* was purchased from Kaiser Pharmaceutical Co., Ltd. (Tainan, Taiwan). Methanol, ethyl acetate and formic acid were obtained from E. Merck (Darmstadt, Germany). Triply deionized water (Millipore, Bedford, MA, USA) was used for all preparations.

### 3.2. Experimental Animals

All experimental protocols involving animals were reviewed and approved by the Institutional Animal Care and Use Committee (IACUC number: 1060813) of National Yang-Ming University. Male Sprague-Dawley rats were obtained from the Laboratory Animal Center of the National Yang-Ming University, Taipei, Taiwan. The animals had free access to food (laboratory rodent diet 5P14, PMI Feeds, Richmond, IN) and water. Six Sprague-Dawley rats (220–240 g) were anesthetized with pentobarbital (50 mg/kg, i.p.) for cannulation. Surgical sites were shaved and disinfected with 70% ethanol solution, and a polyethylene tube (PE50) was implanted into the right jugular vein for collecting blood samples. The cannula were exteriorized and fixed in the dorsal region of the neck. Patency of the tube was maintained by flushing with heparinized saline (20 IU/mL). Rats were allowed to recover 24 h prior to drug administration. Schizandrin was dissolved in 10%DMSO and triply deionized water and administered by gastric gavage at a dose of 10 mg/kg. Schizandrin was dissolved in 10%ethanoland triply deionized water and administered by intravenous injection at a dose of 10 mg/kg. The *S. chinensis* pharmaceutical product was dissolved in triply deionized water and administered by gastric gavage at a dose of 3 g/kg and 10 g/kg (equivalent to 5.2 mg/kg and 17.3 mg/kg schizandrin).The gavage administration of *S. chinensis* volume was 3 mL. A 200 μL blood sample was withdrawn from the right jugular vein at the carotid artery into a heparin-rinsed vial at 5, 15, 30, 45, 60, 90, 120, 150, 180, 240, 360 and 480 min. Blood samples were centrifuged at 6000× *g* for 10 min at 4 °C. The biological samples were stored at −20 °C before analysis.

### 3.3. Sample Preparation

Each blood sample (50 μL) was mixed with 1 mL of ethyl acetate and vortexed for 5 min. The mixture was centrifuged at 16000× *g* for 10 min at 4 °C. The supernatant (1 mL) was transferred to an Eppendorf vial and dried at 40 °C in a centrifugation evaporator. The dried sample was reconstituted in 190 μL of methanol and IS solution (methyl yellow, 5 ng/mL in methanol) and filtered through a 0.22 μm filter. Finally, the filtrate (10 μL) was applied to the UPLC-MS/MS system for analysis.

### 3.4. LC-MS/MS

The LC–MS/MS analysis was performed using a Waters Acquity UPLC™ system (Waters Co., Manchester, UK) consisting of binary solvent manager, an automatic liquid chromatographic sampler and a Waters Xevo^TM^ tandem quadrupole mass spectrometry equipped with an electrospray ionization (ESI) source. The chromatography was performed on a Purospher^®^ STAR RP-18 endcapped (100 mm × 2.1 mm, 2 μm, Merck KGaA, Darmstadt, Germany) analytical column and maintained at 40 °C in a column oven. The mobile phase consisted of 0.1% formic acid-methanol (15:85, *v*/*v*), and the flow rate was set at 0.2 mL/min. The injection volume was 10 μL. The positive ion mode with a multiple reaction monitor (MRM) was used for UPLC-MS/MS analysis. The following precursor-to-product ion transitions were used: *m*/*z* 433.22→415.19 for schizandrin and *m*/*z* 226.26→76.91 for methyl yellow (internal standard, IS).The instrument parameters were optimized as follows: capillary voltage, 3.2 kV; desolvation gas, nitrogen; desolvation temperature, 400 °C; desolvation gas flow rate, 800 L/h; cone voltage, 18 V; collision energy, 12 V and source temperature, 150 °C. MassLynx 4.1 software (Waters Corporation, Milford, MA, USA) was used for data processing.

### 3.5. Method Validation

Method validation was performed based on the US FDA guidelines for bioanalytical method validation (Guidance for Industry: Bioanalytical Method Validation, 2001), which contains methods for the lower limit of quantification, matrix effect, recovery, accuracy and precision, calibration curve and stability.

#### 3.5.1. Accuracy, Precision and Linearity

Linearity was determined in the range of 5–500 ng/mL. The calibration curve was created from the ratio of the peak areas of schizandrin and methyl yellow to the nominal concentration of schizandrin. The correlation coefficient (r^2^) value of all calibration curves was greater than 0.995. The accuracy (bias %) was calculated from the mean value of the observed concentration (C_obs_) and nominal concentration (C_nom_) using the relationship accuracy (bias %) = [(C_obs_ − C_nom_)/C_nom_] × 100. The relative standard deviation (RSD) was calculated from the observed concentrations as precision (RSD %) = [standard deviation (SD)/C_obs_] × 100.Intra-day and inter-day variations of the method in rat samples were less than ±15% (±20% at the lower limit of detection) for all analytes. The limit of quantification (LOQ) was defined as the lowest concentration of the linear range, and the limit of detection (LOD) was defined as the concentration of analyte giving a signal-to-noise ratio (S/N) of 3.

#### 3.5.2. Stability

Stability was evaluated under four conditions: short-term stability kept at room temperature for 6 h; post-preparative stability kept in the autosampler at 8 °C for 6 h; long-term stability kept at −80 °C for 1 month; and freeze-thaw stability: three samples were frozen and stored at −20 °C for 24 h and then thawed at room temperature. After repeating this cycle three times, the samples were analyzed by UHPLC-MS/MS.

#### 3.5.3. Recovery and Matrix Effect

The recovery and matrix effects of schizandrin in various blood samples were assessed at three different concentrations (5, 50, and 500 ng/mL). The methyl yellow (IS) concentration was 5 ng/mL in rat plasma. Three sets of extraction methods were prepared to evaluate recovery and matrix effects in the quantitative bioanalytical method:

*Set 1. Standard schizandrin solutions*. The samples were prepared by placing10 μL of the appropriate concentrations of schizandrin and methyl yellow and 180 μL of methanol into 1.5 mL Eppendorf tubes. After mixing, the solutions were transferred into HPLC vials, and 10 μL was injected into the LC-MS/MS system.

*Set 2. Schizandrin solutions spiked after extraction*. The samples were prepared by placing50 μL of blank plasma in 1.5 mL Eppendorf tubes, followed by the addition of 500 μL of ethyl acetate for a liquid-liquid extract. The samples were mixed (5 min) and centrifuged (15,000 rpm for 10 min at 4 °C) twice. The supernatant was collected in Eppendorf tubes and evaporated to dryness at 50 °C for 30 min and then reconstituted with 10 μL of the appropriate concentration of schizandrin and internal standard and180 µL of methanol. After mixing, the solutions were transferred to HPLC vials, and 10 μL was injected into the LC system for analysis. In set 2, schizandrin was spiked post-extraction into different plasma samples, whereas in set 3, schizandrin was spiked into different plasma samples pre-extraction.

*Set 3. Schizandrin solutions spiked before extraction*. The samples were prepared by placing 50 μL of blank plasma in 1.5 mL Eppendorf tubes, to which 10 μL of appropriate concentrations of schizandrin were added before extraction, followed by the addition of 500 μL of ethyl acetate for liquid-liquid extraction. After centrifugation (15,000 rpm for 10 min at 4 °C), this step was repeated twice. The supernatant was collected in Eppendorf tubes and evaporated to dryness at 50°C for 30 min. Samples were reconstituted with 190 µL of methanol and 10 µL of internal standard. After mixing, the solutions were transferred to HPLC vials, and 10 μL was injected into the LC system for analysis. Recovery (%) and matrix effects (%) were calculated by using the peak area of set 2/set 1 and set 3/set 2, respectively.

### 3.6. Pharmacokinetic Data Analysis

Pharmacokinetic calculations were performed on each individual set of data using the pharmacokinetic software WinNonlin Standard Edition, version 1.1 (Scientific Consulting Inc., Apex, NC, USA) by noncompartmental methods for intravenous and oral administration. The results were analyzed using Student’s t-test and the SigmaPlot program (version 13). All data are presented as the mean ± standard deviation (SD).

## 4. Conclusions

A reliable and sensitive UHPLC-MS/MS method for the analysis of schizandrin in biological samples has been successfully validated and developed. This method had sensitivity, precision, accuracy, selectivity, recovery and stability for the determination of schizandrin in biological samples. In addition, this method was successfully applied in a pharmacokinetic study of schizandrin in freely moving rats after oral administration of pharmaceutical products of *S. chinensis.*

## Figures and Tables

**Table 1:** Method validation for the intra-assay precision (%RSD) and accuracy (%Bias) of the HPLC method for the determination of schizandrin in plasma.

Nominal Concentration (ng/mL)	Observed Concentration (ng/mL)	Precision RSD (%)	Accuracy Bias (%)
Intra-assay			
5	5.01 ± 0.12	1.65	0.19
10	10.22 ± 0.08	2.83	2.16
50	50.23 ± 0.29	0.67	0.45
100	99.55 ± 0.21	0.21	−0.45
500	499.87 ± 0.80	0.16	−0.03
Inter-assay			
5	5.42 ± 0.82	15.16	8.40
10	10.62 ± 0.38	3.55	6.20
50	51.05 ± 2.21	4.33	2.10
100	101.99 ± 2.82	2.76	1.99
500	501.83 ± 2.33	0.46	0.37

The data are presented as the mean ± SD (*n* = 6). RSD: relative standard deviation. Observed concentration data are presented as the mean ± SD. Accuracy (% bias) = [(C_obs_ − C_nom_)/C_nom_] × 100. Precision (%RSD) = [SD/C_obs_] × 100.

**Table 2:** Recovery and matrix effect of schizandrin in rat plasma.

Nominal Concentration (ng/mL)	Peak Areas			Matrix Effect (%)	Recovery (%)
	SET1	SET2	SET3		
Schizandrin					
5	6862 ± 268	6279 ± 682	6118 ± 403	91.5 ± 8.3	97.4 ± 5.8
50	72,483 ± 1267	70,910 ± 3084	70,723 ± 7279	97.8 ± 2.8	99.6 ± 7.1
500	624,413 ± 25,318	572,304 ± 50,936	519,150 ± 31,359	91.6 ± 4.3	90.8 ± 2.7
Average				93.6 ± 5.1	96.1 ± 5.2
Methyl yellow	62,710 ± 2132	72,097 ± 2293	67,947 ± 2912	115.0 ± 4.0	94.4 ± 6.2

The data are presented as the mean ± SD (*n* = 3).

**Table 3:** The stability data for schizandrin in rat plasma.

Blood Concentration (ng/mL)	Short-Term Stability	Autosampler Stability	Freeze-Thaw Stability	Long-Term Stability
5	−1.63 ± 8.36	−1.78 ± 2.25	−8.04 ± 3.19	−0.56 ± 0.39
50	5.70 ± 4.52	3.17 ± 3.19	−5.16 ± 3.83	−9.02 ± 0.40
500	0.64 ± 0.08	0.43 ± 0.12	−0.63 ± 0.30	−6.75 ± 6.39

The data presented as the mean ± SD. Short-term stability: kept at room temperature for 12 h; Autosampler stability: kept at 10 °C for 24 h in autosampler; Freeze-thaw stability: three freeze-thaw cycles; Long-term stability: kept at −20 °C for 1 month.

**Table 4:** Pharmacokinetic parameters of schizandrin in rat plasma.

Parameter	Schizandrin		*Schisandra chinensis*	
	10 mg/kg, i.v.	10 mg/kg, p.o.	3 g/kg, p.o.	10 g/kg, p.o.
C_max_ (µg/mL)		0.06 ± 0.03	0.08 ± 0.07	0.15 ± 0.09 ^a^
AUC (min ng/mL)	43.11 ± 5.62	6.71 ± 4.51	17.58 ± 12.31	28.03 ± 14.29 ^a^
t_1/2_ (min)	42.25 ± 14.84	74.69 ± 33.56	68.20 ± 23.93	51.97 ± 12.35
T_max_ (min)		22.50 ± 12.55	185.00 ± 101.14 ^a^	200.00 ± 45.17 ^a^
CL (L/min/kg)	0.24 ± 0.03	0.34 ± 1.24	0.33 ± 0.24	0.30 ± 0.46
MRT (min)	34.80 ± 7.53	113.42 ± 41.65	206.07 ± 41.97 ^a^	213.63 ± 32.00 ^a^
F (%)		15.56 ± 10.47	78.42 ± 54.91 ^a^	37.59 ± 19.16 ^a^

Data are presented as the mean ± SD, *n* = 6. AUC: area under the concentration-time curve; CL: clearance; MRT: mean residence time; F, bioavailability. The dose of *S. chinensis* (3 g/kg) equivalent to schizandrin (5.2 mg/kg); the dose of *S. chinensis* (10 g/kg) equivalent to schizandrin (17.3 mg/kg). ^a^ Significantly different from oral ingestion schizandrin group at *p* < 0.05.
